# Effects of Developmental Activation of the Aryl Hydrocarbon Receptor by 2,3,7,8-Tetrachlorodibenzo-*p*-dioxin on Long-term Self-renewal of Murine Hematopoietic Stem Cells

**DOI:** 10.1289/ehp.1509820

**Published:** 2015-10-23

**Authors:** Michael D. Laiosa, Everett R. Tate, Lori S. Ahrenhoerster, Yuhong Chen, Demin Wang

**Affiliations:** 1 Joseph J. Zilber School of Public Health, University of Wisconsin–Milwaukee, Milwaukee, Wisconsin, USA; 2 Blood Research Institute, BloodCenter of Wisconsin, Milwaukee, Wisconsin, USA

## Abstract

**Background:**

Human epidemiological and animal studies suggest that developmental exposure to contaminants that activate the aryl hydrocarbon receptor (AHR) lead to suppression of immune system function throughout life. The persistence of immune deficiency throughout life suggests that the cellular target of AHR activation is a fetal hematopoietic progenitor or stem cell.

**Objectives:**

The aim of this study was to identify the effects of transplacental exposure to an AHR agonist on long-term self-renewal of fetal hematopoietic stem cells.

**Methods:**

Pregnant C57BL/6 or AHR+/– mice were exposed to the AHR agonist, 2,3,7,8-tetra-​chlorodibenzo-p-dioxin (TCDD). On day 14 of gestation, hematopoietic progenitors from wild-type or AHR-deficient fetuses were placed into in vitro T-lymphocyte differentiation cultures to identify the effects of transplacental TCDD on AHR activation in the fetus. We next analyzed the fetal hematopoietic progenitor cells for changes in reactive oxygen species (ROS). Finally, hematopoietic progenitors from fetuses exposed transplacentally to TCDD were mixed 1:1 with cells from congenic controls and used to reconstitute lethally irradiated recipients for analysis of long-term self-renewal potential.

**Results:**

Our findings suggested that the effects of TCDD on the developing hematopoietic system were mediated by direct AHR activation in the fetus. Furthermore, developmental AHR activation by TCDD increased ROS in the fetal hematopoietic stem cells, and the elevated ROS was associated with a reduced capacity of the TCDD-exposed fetal cells to compete with control cells in a mixed competitive irradiation/reconstitution assay.

**Conclusions:**

Our findings indicate that AHR activation by TCDD in the fetus during pregnancy leads to impairment of long-term self-renewal of hematopoietic stem cells.

**Citation:**

Laiosa MD, Tate ER, Ahrenhoerster LS, Chen Y, Wang D. 2016. Effects of developmental activation of the aryl hydrocarbon receptor by 2,3,7,8-tetrachlorodibenzo-p-dioxin on long-term self-renewal of murine hematopoietic stem cells. Environ Health Perspect 124:957–965; http://dx.doi.org/10.1289/ehp.1509820

## Introduction

Since nutritional deficiency during pregnancy was first identified as a fundamental factor in the developmental origins of health and disease, there has been an explosion in the number of factors that have been identified as influencing lifetime health status for nearly every organ system in the body (Barker 2007; Haugen et al. 2015). Moreover, intrauterine environmental factors such as exogenous chemicals are clearly recognized to increase the risk for a spectrum of disorders that may appear during childhood and that can persist throughout life (Faulk and Dolinoy 2011; Haugen et al. 2015). Immune system function has been recognized as a particularly sensitive end point to changes in the intrauterine environment owing to its systemic distribution throughout the body and its importance for both host defense and immunoregulatory function (Dietert 2011; Winans et al. 2011). Specifically, human epidemiological studies have found associations between developmental exposures and an array of later-life immune deficiencies including changes in cord blood lymphocyte composition, increased wheezing events, and increased autoinflammatory disorders (Choi et al. 2010; Herr et al. 2010; Jedrychowski et al. 2014). Additionally, animal studies have found that transplacental exposures to chemicals that bind to and activate the aryl hydrocarbon receptor (AHR) adversely affect later-life immune effects by decreasing the immune response to influenza and by increasing autoimmune susceptibility in adults (Boule et al. 2014; Hogaboam et al. 2008; Mustafa et al. 2009). The diversity of adult diseases caused by related developmental exposures may be consistent with a two-hit mechanism whereby the prenatal exposure leads to epigenetic reprogramming of a progenitor cell that can have differential impacts on disease etiology depending on the genetic background, timing, and type of secondary environmental exposures. Indeed, this added complexity to the developmental origins of health and disease hypothesis has been proposed for other adult outcomes including neurodevelopmental, reproductive, and obesegenic disorders (Bruner-Tran and Osteen 2011; Lahiri and Maloney 2010; Wadhwa et al. 2009).

The importance of the AHR to human health is demonstrated in part by epidemiological studies based on the population in Seveso, Italy, that was accidentally exposed to the prototypical AHR agonist 2,3,7,8-tetra-​chlorodibenzo*-p*-dioxin (TCDD), where the risk for lymphatic and hematological cancers was found to be slightly elevated in adults (Consonni et al. 2008; Pesatori et al. 2009). Furthermore, U.S. Air Force veterans serving during the Vietnam War who were exposed to TCDD-contaminated Agent Orange were reported to be at greater risk for melanoma and prostate cancer than unexposed veterans (Akhtar et al. 2004). In both the Seveso cohort and among the Vietnam veterans, small sample sizes for relatively rare diseases have made it difficult to establish causal conclusions between TCDD exposure and disease. Nevertheless, the International Agency for Research on Cancer (IARC) has classified TCDD as a Group I human carcinogen based on sufficient epidemiological evidence for all cancers combined (Baan et al. 2009). Despite residual uncertainty regarding the carcinogenicity of TCDD in the general population, there is a need to identify and understand intergenerational impacts of dioxins given the unique vulnerability of children to developmental exposures (Landrigan and Goldman 2011). Notably, low-level background exposures to chemicals that activate the AHR occur primarily through dietary intake (Kvalem et al. 2012; Liem et al. 2000; Schecter et al. 2001). Furthermore, human developmental evidence specific to the immune system has been obtained from epidemiological studies that found an association between prenatal exposure to dioxins and dioxin-like PCBs and lower antibody titers for mumps and measles at preschool age (Weisglas-Kuperus et al. 2000).

Given the known risk for immune system impairment from developmental exposure to compounds that activate the AHR (Winans et al. 2011), it remains critical to identify the long-term cellular and molecular targets in the hematopoietic system. In experimental systems, the AHR has been identified as a negative regulator of hematopoietic stem cell proliferation (Singh et al. 2014). A study of mice exposed to TCDD as adults demonstrated that AHR activation had adverse effects on long-term self-renewal (Sakai et al. 2003). Furthermore, a transient inhibition of prothymocyte activity in fetal hematopoietic progenitors was reported following developmental TCDD exposure in mice (Fine et al. 1989). Despite a growing consensus that the AHR is an important regulator of hematopoiesis (Boitano et al. 2010; Carlin et al. 2013; Casado et al. 2011; Smith et al. 2013), the impact of activation of the AHR in the fetus on the long-term self-renewal function of the hematopoietic system is entirely unknown.

To address this question, pregnant mice were exposed to a low oral dose of TCDD, and we first tested the specific role of the AHR in the fetus on the differentiation potential of fetal hematopoietic progenitors. We found that developmental TCDD exposure increased the fluorescence of an indicator dye that measures cellular reactive oxygen species (ROS) in fetal hematopoietic progenitor and stem cells. Using ROS as a marker of hematopoietic stem cell differentiation, we then sorted cells for gene expression analysis and found differential expression of Notch pathway genes in TCDD-exposed fetuses. Finally, we performed an irradiation/competitive reconstitution experiment using fetal liver progenitor cells from vehicle-exposed fetuses and fetuses that had been exposed to TCDD transplacentally. We found that cells derived from the TCDD-exposed fetuses were significantly reduced in all immune tissues when competing against hematopoietic progenitors from the controls.

## Materials and Methods

### Experimental Animals

All animal procedures were conducted humanely and with regard for the alleviation of pain and suffering according to NIH’s Guide for the Care and Use of Laboratory Animals [National Research Council (U.S.) Committee for the Update of the Guide for the Care and Use of Laboratory Animals (NRC) 2011] and with the approval of the Institutional Animal Care and Use Committees (IACUC) at the University of Wisconsin-Milwaukee and the Blood Research Institute of Wisconsin. C57BL/6J mice, designated CD45.2, or B6.SJL-Ptp^rca^ Pepc^b^/BoyJ mice, designated CD45.1, were offspring from original breeder pairs obtained from the Jackson Laboratory. B6;129-Ahr^tm1Bra^/J mice, henceforth designated AHR^+/–^, were obtained from the Jackson Laboratory and bred as female heterozygotes by male AHR^–/–^. After overnight pairings, the presence of a vaginal plug was designated gestational day (GD) 0.5. All mice were housed in Ancare 75 high-temperature polycarbonate microisolator cages [11.75 in × 7.25 in × 5 in; 75 in^2^ (19.05 cm × 29.21 cm × 12.7 cm; 483.87 cm^2^)] with ¼ in of Harlan Teklad corncob bedding in a specified pathogen-free facility at the University of Wisconsin-Milwaukee. No more than five adult mice were housed per cage, and reproductively active males were housed individually. Mice were provided with Teklad Irradiated Global 19% Protein Extruded Rodent Diet 2919 and autoclaved tap water *ad libitum.* Mice were maintained on a 12:12-hr light:dark cycle, and room temperature was maintained at 22°C ± 1.5°C. Unless otherwise specified, four to six mice were used per group for each experiment.

### TCDD Preparation and Treatment Protocol

TCDD (Cambride Isotopes) diluted in 1,4-dioxane (Sigma-Aldrich) at a working stock concentration of 0.2 mg/mL was suspended in olive oil (Filippo Berio) to a concentration of 0.3 μg/mL as previously described (Ahrenhoerster et al. 2014). The vehicle control was prepared with an equal volume of evaporated 1,4-dioxane added to olive oil. Timed pregnant mice were exposed to TCDD at a dose of 3 μg/kg body weight or to olive oil vehicle (0.1 mL per 10 g) by oral gavage on gestational days 0.5 and 7.5.

### Antibodies for Lymphocyte Staining

Primary fluorochrome-conjugated monoclonal antibodies were used in flow cytometry analysis and cell sorting. All antibodies were used at titrated concentrations and were purchased from BD Biosciences unless otherwise noted. Biotin-conjugated antibodies used for the lineage cocktail included CD3 (clone 145-2c11), LY-76 (clone TER119), CD45R/B220 (clone RA3-6B2), CD11b (clone M1/70), and LY6G/LY6C/GR-1 (clone RB6-8C5) coupled with streptavidin–fluorescein isothiocyanate (streptavidin-FITC). Lineage-negative cells were further identified with phycoerythrin (PE)-conjugated Sca1 (clone E13-161.7) and Alexa647-conjugated c-Kit (clone 2B8; Life Technologies). Thymocytes and splenocytes were identified with Alexa647-conjugated CD8α (clone 53-6.7), PE-conjugated CD4 (clone L3T4), and APC-H7-conjugated B220 (clone RA3-6B2). FITC-conjugated CD45.1 (clone NDS58) and PE-Cy7-conjugated CD45.2 (clone 104) were used to identify chimerism within the bone marrow, thymus, and spleen.

For analysis of relative intracellular ROS levels, cells were first stained for cell surface proteins and then incubated with 5 μM 2´7´-dichloroflourescein diacetate (H_2_DCF-DA; Life Technologies) in phosphate-buffered saline (PBS) containing 5% fetal bovine serum. H_2_-DCF-DA is hydrolyzed by intracellular reactive oxygen species into highly fluorescent DCF, which is detectable in the FITC channel of the BD FACSAria III cell sorter (BD Biosciences). After a 15-min incubation in a 37°C water bath, cells were then acquired on the BD FACSAria III.

To measure apoptosis, fetal progenitor cells from GD 14.5 were stained to identify LSK cells (defined by an absence of lineage markers and the expression of Sca1 and c-Kit), fixed, and permeabilized; DNA breakage was analyzed by the terminal deoxynucleotidyl transferase (TdT)-dependent nick-end labeling (TUNEL) assay using an APO-BrdU kit (Phoenix Flow Systems) according to the manufacturer’s instructions.

### Isolation of Fetal Liver Hematopoietic Progenitors for Limiting Dilution Analysis

Pregnant mice were euthanized by CO_2_ asphyxiation followed by cervical dislocation to confirm death in accordance with the American Veterinary Medical Association (AVMA) Guidelines on Euthanasia (Leary et al. 2013) on gestational day 11.5 or 14.5. For all experiments, mice were euthanized between 0730 and 0900 hours in our laboratory, which is inspected twice per year by the UW-Milwaukee IACUC. Fetal livers were dissected and cell suspensions were prepared and used for limiting dilution analysis with exactly 1, 3, 10, or 30 LSK cells directly sorted into individual wells on a Costar tissue culture–treated 96-well plate (Corning) containing mitomycin-C treated OP9-DL1 stromal cells grown to confluence as previously described (Ahrenhoerster et al. 2014). The limiting dilution assays testing the role of the AHR in TCDD responsiveness were accomplished by breeding AHR^–/–^ male mice with AHR^+/–^ females such that the dams and half the fetuses would be responsive to TCDD. In comparison, the AHR^–/–^ fetuses were anticipated to be resistant to the direct effects of *in utero* TCDD exposure. Cell sorting was performed using a BD FACSAria III, DIVA v.6.1.3, equipped with four lasers (violet 405 nm, blue 488 nm, yellow/green 561 nm and red 633 nm), four-way sorting capacity, and an automated cell deposition unit capable of sorting a single cell into an individual well.

### Competitive Irradiation Chimeras

On GD 14.5, fetal liver hematopoietic progenitors were harvested for the limiting dilution experiment as described above. After depletion of red blood cells (RBCs), cells from vehicle-exposed CD45.1 fetuses were counted and mixed with an equivalent number of fetal liver cells from the vehicle- or TCDD-exposed CD45.2^+^ dams.

Four hours before reconstitution, host CD45.1^+^ mice were lethally irradiated with 11 Gy to eliminate all host hematopoietic cells. Subsequently, 1,000,000 fetal liver cells (500,000 CD45.1 cells plus 500,000 CD45.2 cells) were injected into each host recipient mouse by intravenous (IV) injection. Mice were then maintained for 8 weeks to allow complete blood system reconstitution. At 8 weeks, the primary competitive chimera recipients were euthanized by CO_2_ asphyxiation followed by cervical dislocation as described above, with the exception that the procedure was performed at the Blood Research Institute between 0900 and 1000 hours. Bone marrow obtained from one femur as well as the thymus and the spleen were analyzed for the percent of chimerism. Bone marrow from the other femur was harvested under sterile conditions, depleted of RBCs, and counted, and 1 × 10^6^ cells were used in a secondary IV transfer into individual naïve CD45.1^+^ irradiated host recipient mice. Transfers into the secondary recipients were performed by IV injection of cells from individual mice into each individual recipient to track any potential outliers throughout the full 16-week experiment. Eight weeks after the transfer, the secondary recipients were euthanized, and lymphoid tissues were analyzed for chimerism as with the primary recipients.

For analysis of competitive mixed chimeras, ≥ 100,000 viable lymphocytes from bone marrow, thymus, or spleen were acquired, and chimerism was analyzed by comparing the frequency of CD45.1 and CD45.2. Additional analysis of cell subset distribution was determined by first gating on CD45.1 or CD45.2, then analyzing lineage markers Sca1 and c-Kit (bone marrow) or CD4, CD8, and B220 (thymus and spleen).

### RNA Preparation and Quantitative RT-PCR

On GD 11.5, hematopoietic cells pooled from individual litters of vehicle- or TCDD-exposed fetuses were isolated and sorted directly into Trizol (Life Technologies) using c-Kit and DCF fluorescence to discriminate between hematopoietic progenitor cells with long- and short-term self-renewal potential, respectively. RNA was purified, quantified and processed as previously described (Ahrenhoerster et al. 2014). RNA was subjected to reverse transcription using a Tetro cDNA Synthesis kit (Bioline) with both anchored-oligo(dT) 18 priming and random hexamer priming options and was stored at –20°C until the day of the assay. Primers were selected using Universal ProbeLibrary v.2.5 for Mouse (Roche) and checked for specificity using Primer-BLAST (http://www.ncbi.nlm.nih.gov/tools/primer-blast/). Gene names, accession numbers, and primer sequences are provided in Table S1. cDNA was used as a template in a 20-μL reaction consisting of 10 μmol of forward and reverse primers and 10 μL SensiFAST SYBR No-ROX (Bioline). Relative expression change was determined using the 2^–ΔΔC_T_^ method with standardization to housekeeping genes. Samples were run in triplicate wells with at least two independent litters per treatment analyzed. Cycling conditions were 95°C for 2 min, followed by 45 cycles at 95°C for 5 sec, 60°C for 10 sec, and 72°C for 10 sec.

### Statistical Analysis

**Figure 1:**
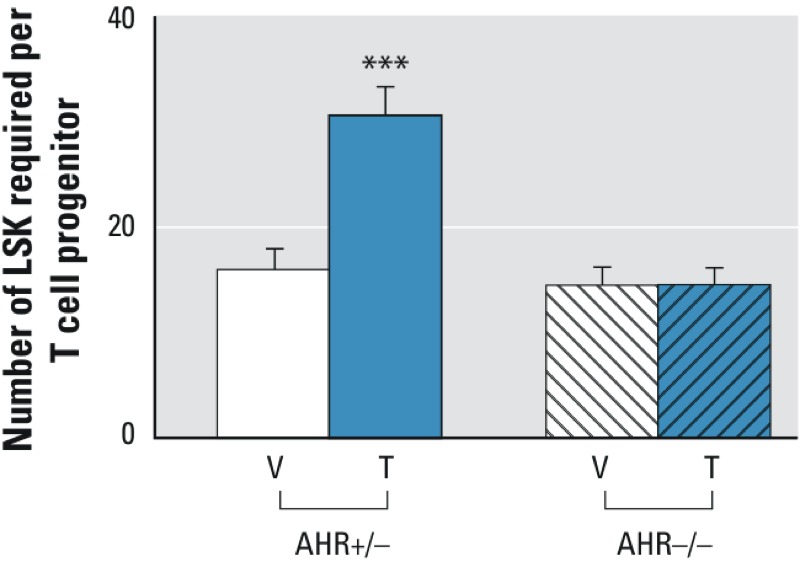
Effects of developmental ­tetrachlorodibenzo*-­p*-dioxin (TCDD) exposure and fetal aryl hydrocarbon receptor (AHR) expression on the capacity of hemato­poietic progenitor cells to undergo ­T-­lymphocyte differentiation. Fetal liver hemato­poietic stem cells from gestational day (GD) 14.5 fetuses were placed into OP9-DL1 limiting dilution *in vitro* coculture experiments. Twelve days later, hematopoietic stem cell differentiation into thymocytes was determined as a measure of ­T-­cell differentiation potential. Data are reported as the average number of hematopoietic stem cells placed into culture needed to produce a single thymocyte colony. Error bars represent the upper bound of the 95% confidence interval for each group. Data from AHR^+/–^ fetuses are represented by solid bars, and data from AHR^–/–^ fetuses are represented by bars containing diagonal slashes. The white bars represent fetuses from dams exposed to vehicle, whereas the blue bars are from ­TCDD-­exposed fetuses. ***p ≤ 0.01 by ­chi-­squared analysis comparing ­TCDD-­exposed AHR^+/–^ fetuses to ­vehicle-­exposed AHR^+/–^ fetuses. Data are pooled from three independent experiments.

The T-cell precursor frequency calculations and chi-squared statistical analysis for each genotype and exposure reported in Figure 1 were accomplished using the “limdil” function contained in the “statmod” package in Rstudio as previously described (RStudio 2012; Ahrenhoerster et al. 2014).

**Figure 2:**
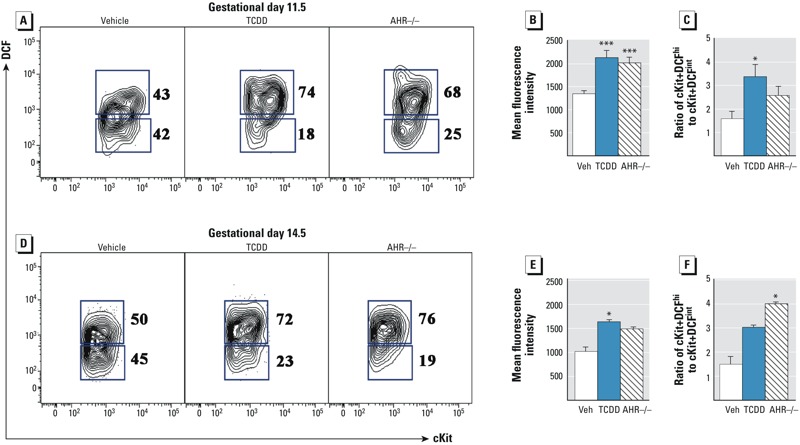
Effects of developmental ­tetrachlorodibenzo*-­p*-dioxin (TCDD) exposure and fetal aryl hydrocarbon receptor (AHR) expression on reactive oxygen species in fetal hematopoietic progenitors. For the analysis shown in Figure 2, viable ­lymphocyte-­sized cells were electronically gated based on ­lineage-­c-Kit^+^ as illustrated in Figure S1. (*A*) Representative ­c-­Kit versus dichlorofluorescein (DCF) flow cytometry plots on gestational day (GD) 11.5 for vehicle (Veh), TCDD, and AHR^–/–^. The number to the right of each ­c-­Kit^+^DCF gate indicates the percentage of cells within each population. (*B*) Mean fluorescence intensity of total DCF in ­c-­Kit^+^ cells. (*C*) The percentage of GD 11.5 ­c-­Kit^+^ cells in the DCF^hi^ gate is compared with the percentage of cells in the DCF^int^ gate to illustrate that TCDD both increased the overall DCF profile and changed the distribution of the cells within the bimodal distribution. (*D*) On GD 14.5, ­li^n^–­c-­Kit^+^Sca1^+^ (LSK) cells were further analyzed for ­c-­Kit versus DCF as shown in Figure 2A. (*E*) Mean fluorescence intensity of total DCF in LSK cells on GD 14.5. (*F*) Ratio of DCF^hi^ cells to DCF^int^ cells in the GD 14.5 LSK population. Data in the bar graphs are the mean ± SEM with *n* = 8 individual fetuses per C57BL/6 group from two separate litters and 4 AHR^–/–^ fetuses. The experiment was repeated twice. Statistical significance determined by Tukey’s *­*t-­test after analysis of variance (ANOVA) is denoted with * for *p* ≤ 0.5 or *** for *p* ≤ 0.01 compared with vehicle.

**Figure 3:**
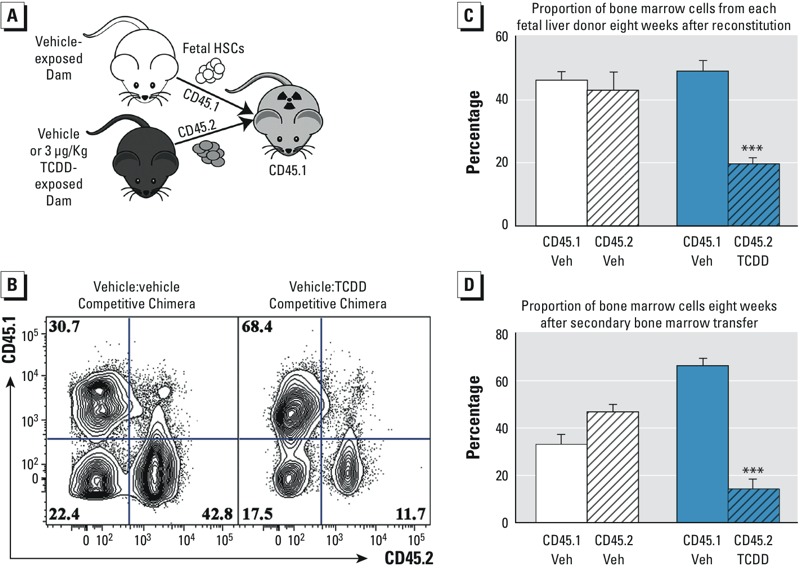
Effects of transplacental ­tetrachlorodibenzo*-­p*-dioxin (TCDD) exposure on the ­long-­term ­self-­renewal potential of fetal liver hematopoietic stem cells. (*A*) Schematic model of the experimental design for the primary reconstitution experiment. (*B*) Representative flow cytometry plot of bone marrow from the primary chimera. Numbers in each quadrant of the flow cytometry plots represent the percentage of bone marrow cells identified by the antibody specific for CD45.1 or CD45.2 congenic surface proteins. (*C*) Percent of bone marrow cells from each donor in the primary and secondary recipients. White bars represent control competitive chimeras, and blue bars represent the chimeras where vehicle (Veh) cells were competed with cells obtained from ­TCDD-­exposed fetuses. Solid bars represent CD45.1^+^ cells, and CD45.2^+^ cells are denoted with diagonal ­slash-­filled bars. Data are the mean ± SEM with 5 mice per group. The experiment was repeated twice. (*D*) Percent of bone marrow cells from each donor after the secondary bone marrow transfer. *** Indicates statistical significance by analysis of variance (ANOVA) followed by Tukey’s test; *p *< 0.01 compared with the congenic cells from the same chimera.

**Figure 4:**
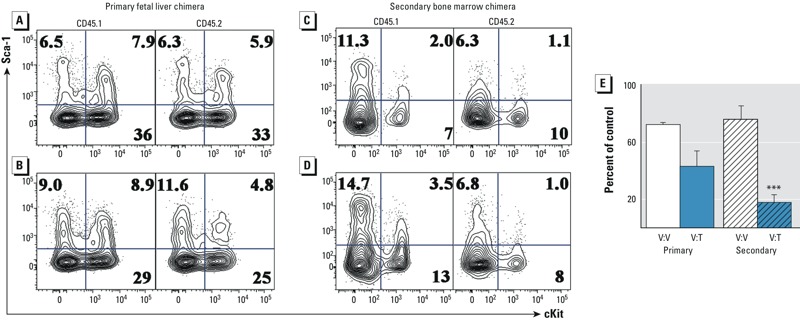
Effects of developmental ­tetrachlorodibenzo*-­p*-dioxin (TCDD) exposure on bone marrow hematopoietic progenitor cells after ­long-­term competitive reconstitution. (*A*) ­c-­Kit versus Sca1 flow cytometry plots from the primary ­vehicle-­to-vehicle (V:V) competitive chimeras with CD45.1^+^lin^–^ cells in the left panel and CD45.2^+^lin^–^ cells in the right panel. The percentage of lin^–^ cells is specified by the number in each quadrant. (*B*) Flow cytometry plots from the primary vehicle to TCDD competitive chimeras. (*C*) Representative ­c-­Kit versus Sca1 flow cytometry plots from the ­vehicle-­to-vehicle secondary bone marrow chimera. (*D*) Flow cytometry plots from the secondary vehicle to TCDD (V:T) competitive chimeras. (*E*) Relative frequency of ­li^n^–­c-­Kit^+^Sca1^+^ (LSK) hematopoietic progenitor cells in the CD45.2 population of cells compared with the CD45.1 competitor cells from the primary and secondary chimeras. White bars compare ­vehicle-­to-vehicle chimeras, and blue bars compare the vehicle with TCDD chimeras. Solid bars are from the primary chimeras, and secondary chimeras are represented by bars containing slashes. The percentage of the control was calculated by dividing the frequency of ­c-­Kit^+^Sca1^+^ cells in the CD45.2^+^lin^–^ population by the same ­population of CD45.1^+^lin^–^ cells. Data are the mean ± SEM from *n* = 5 chimeras, and the experiment was repeated once. *** Denotes statistically significant by analysis of variance (ANOVA) followed by Tukey’s test; *p *< 0.01 compared with the congenic cells from the same chimera.

**Figure 5:**
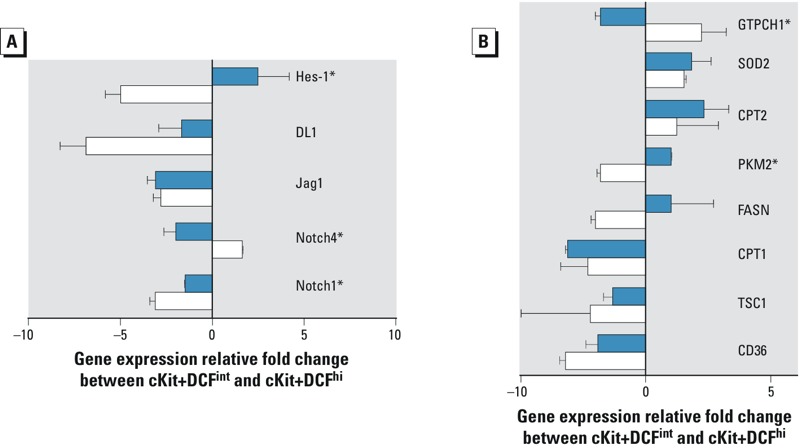
Impact of developmental ­tetrachlorodibenzo*-­p*-dioxin (TCDD) exposure on ­differentiation-­induced changes in gene expression in hematopoietic progenitor cells. Hematopoietic stem and progenitor cells were purified by fluorescence activated cell sorting as illustrated in Figure 2A and with the sorting strategy shown in Figure S4. (*A*) Gene expression changes associated with Notch signal transduction between ­c-­Kit^+^DCF^int^ and ­c-­Kit^+^DCF^hi^ cells from control fetuses (white bars) and ­TCDD-­exposed fetuses (blue bars). (*B*) Gene expression changes associated with cellular metabolism (CD36, TSC1, CPT1, CPT2, FASN, PKM2) and oxidative stress (SOD2, GTPCH1) in ­c-­Kit^+^DCF^int^ and ­c-­Kit^+^DCF^hi^ cells from control fetuses (white bars) and ­TCDD-­exposed fetuses (blue bars). Data are presented as the mean ± standard deviation of replicate wells from a single quantitative polymerase chain reaction (qPCR) reaction with the experiment performed on two independent litters of mice. *p ≤ 0.05, statistical significance between the two treatments (Student’s *­*t-­test).

GraphPad Prism (GraphPad Software) was used for the statistical analysis and graphical presentation of all other data. Specifically, GraphPad was utilized to perform the analysis of variance and post hoc Tukey’s tests on the data obtained in the competitive reconstitution chimera experiments and the analysis of DCF fluorescence (Figures 2, 3, 4). The statistical significance of changes in gene expression was determined by Student’s t-test (Figure 5). For all experiments, *p* ≤ 0.05 indicated statistical significance.

## Results

### Effects of Developmental TCDD Exposure on T-Lymphocyte Differentiation Capacity of Hematopoietic Progenitor Cells from AHR-Deficient or AHR^+/–^ Fetuses

Adverse effects of developmental exposures may be directly mediated by the toxicant in the developing fetus, or they may be mediated via a toxic response occurring in the mother that adversely affects the endocrine and nutrient-exchange systems required for growth of the fetus. To determine whether the fetal or the maternal environment is the dominant target of TCDD in the developing hematopoietic system, we tested the T-lymphocyte differentiation capacity of hematopoietic progenitor cells obtained from AHR^+/–^ or AHR^–/–^ fetuses from pregnant dams exposed to vehicle or to 3 μg/kg TCDD. T-lymphocyte differentiation capacity was assessed using the OP9-DL1 *ex vivo* assay that has previously been employed to demonstrate that developmental exposure to TCDD from GD 0.5 through GD 14.5 attenuates the capacity of fetal hematopoietic progenitor cells to complete lymphocyte differentiation (Ahrenhoerster et al. 2014). Indeed, as shown in Figure 1, only hematopoietic progenitors obtained from TCDD-exposed AHR^+/–^ fetuses had an attenuated T-lymphocyte differentiation potential. Specifically, the T-cell progenitor frequency of hematopoietic progenitors was nearly identical in all but the TCDD-exposed AHR^+/–^ cells, with a progenitor frequency of 1 in 15.6 (95% CI: 13.6, 18.0) for vehicle-exposed AHR^+/–^, 1 in 14.2 (95% CI: 12.4, 16.3) for vehicle-exposed AHR^–/–^ cells, and 1 in 14.3 (95% CI: 12.7, 16.1) for TCDD-exposed AHR^–/–^ cells. In comparison, the T-cell progenitor frequency of LSK cells from TCDD-exposed AHR^+/–^ hematopoietic progenitor cells was 1 in 30.4 (95% CI: 27.7, 33.5). These data indicate that the pool of hematopoietic progenitor cells from TCDD-exposed AHR^+/–^ fetuses have an approximate 2-fold decrease in T-lymphocyte differentiation potential compared with vehicle and TCDD-exposed AHR^–/–^ fetuses.

### Effects of Developmental TCDD Exposure on Reactive Oxygen Species in Fetal Hematopoietic Progenitor Cells

Given the deleterious effects of developmental AHR activation on T-cell differentiation, it was necessary to probe more specifically the function of the hematopoietic stem and progenitor cell pools in the fetus. As an initial approach to testing this function, we assessed the levels of intracellular reactive oxygen species (ROS) following developmental exposure to TCDD. We analyzed ROS because hematopoietic stem and progenitor cells maintain a reduced basal metabolic rate, thereby preventing accumulation of DNA-mutagenic ROS that could affect multilineage differentiation and long-term self-renewal (Ito et al. 2006; Jang and Sharkis 2007). Intracellular ROS were detected in hematopoietic stem and progenitor cells from GD 11.5 and 14.5 fetuses by the esterified vital dye H_2_DCF-DA, which is oxidized to highly fluorescent DCF in the presence of oxidative stress. The GD 14.5 time point was chosen to identify potential cellular changes occurring in the cells that coincided with the time point used in the differentiation experiment shown in Figure 1. In comparison, GD 11.5 cells were initially analyzed to characterize the hematopoietic stem cells before the differentiation potential was interrogated. Furthermore, GD 11.5 represents an important transition in fetal hematopoiesis; at this point, hematopoietic progenitors are beginning to be defined as true stem cells because of their acquisition of long-term self-renewal (Ciriza et al. 2013; North et al. 2002). As shown in Figure S1, the DCF fluorescence changed depending on the specific cell population that was analyzed; thus, it was necessary to analyze DCF specifically within the putative hematopoietic progenitor and stem cell populations. Furthermore, we analyzed DCF in progenitor cells from age-matched AHR^–/–^ fetuses given previous reports that ROS were elevated in bone marrow hematopoietic stem cells from AHR^–/–^ mice (Singh et al. 2014). As shown in Figure 2A, there was a bimodal distribution of DCF within the c-Kit^+^ hematopoietic progenitor cells on GD 11.5. Thus, an electronic gate surrounding each of the two DCF populations was made using the vehicle sample, and the populations were designated c-Kit^+^DCF^hi^ (top gate in Figure 2A) and c-Kit^+^DCF^int^ (lower gate in Figure 2A). Notably, total DCF fluorescence was clearly elevated in the cells from TCDD-exposed fetuses, as indicated by the population shift toward the upper gate in the plot at the far right of Figure 2A. In AHR^–/–^ fetuses, the basal level of DCF fluorescence in c-Kit^+^ progenitors is nearly the same as that observed in the TCDD-exposed fetuses. Specifically, developmental AHR activation by TCDD in C57Bl/6 mice accompanied by absence of the AHR increased the overall fluorescence intensity of DCF by nearly 75%, indicating higher levels of intracellular ROS in fetal hematopoietic stem and progenitor cells (Figure 2B). Moreover, by taking a ratio of the percentage of c-Kit^+^DCF^hi^ cells to the c-Kit^+^DCF^int^, it is evident that developmental AHR activation increased the distribution of hematopoietic progenitors with high intracellular ROS relative to progenitors with a lower intracellular oxidative state (*p* ≤ 0.05) (Figure 2C). The elevated ROS was not associated with an increase in apoptosis in hematopoietic progenitors as measured by TUNEL assay (see Figure S2). Similar effects of developmental AHR activation on ROS were observed on GD 14.5 (Figure 2D); however, the magnitude of change in the DCF fluorescence was less pronounced than on GD 11.5 (Figure 2E). Despite the slightly lower basal ROS at GD 14.5 than at GD 11.5, there was a slight, but not statistically significant, increase in the ratio of c-Kit^+^DCF^hi^ cells to c-Kit^+^DCF^int^ cells in progenitors from TCDD-exposed fetuses on GD 14.5. In comparison, there was a statistically significant increase in this ratio in AHR^–/–^ fetuses (*p* ≤ 0.05; Figure 2F).

### Effects of Developmental TCDD Exposure on Long-Term Self-Renewal Capacity of Hematopoietic Stem Cells

Self-renewal is a fundamental property of hematopoietic stem cells; this term refers to the principle that each time a stem cell divides, one of the daughter cells will replace the functions of the original parent cell while the other cell undergoes multilineage differentiation. Testing the functional capacity of a hematopoietic stem cell pool to maintain long-term self-renewal potential is accomplished by mixing wild-type control progenitor cells with a test population and following the ability of each population to sustain hematopoiesis in a lethally irradiated host (Richie Ehrlich et al. 2011; Weissman 2000). As illustrated in Figure 3A, we performed this competitive reconstitution experiment by competing GD 14.5 fetal liver cells from vehicle-exposed CD45.1 fetuses with CD45.2 fetal liver cells from either vehicle-exposed or TCDD-exposed dams. Eight weeks after the initial reconstitution, the contribution of each donor pool was analyzed in the bone marrow, thymus, and spleen of the initial recipients. Furthermore, bone marrow from each recipient was transferred into a secondary recipient to confirm the long-term self-renewal potential of each donor pool. We found that the hematopoietic stem cell pool obtained from the CD45.2^+^ TCDD-exposed fetuses failed to compete effectively over the course of the primary and secondary irradiation/reconstitution procedures (Figure 3B). Notably, the diminished competitive capacity of the hematopoietic stem cells from the TCDD-exposed fetuses could be observed after the initial reconstitution, when the cells from the control exceeded the TCDD by nearly 152% (Figure 3C, *p* ≤ 0.01). This difference was further exacerbated following the secondary transfer, when the control cells outcompeted the TCDD-exposed hematopoietic progenitors by approximately 381% (Figure 3D, *p* ≤ 0.01).

To determine whether the diminished competitive capacity of TCDD-exposed fetal progenitors was reflected by a reduction in hematopoietic stem and progenitor cells in the reconstituted primary and secondary bone marrow, we analyzed the frequency of CD45.1- and CD45.2-derived hematopoietic stem and progenitor cells, defined by an absence of lineage markers and the expression of Sca1 and c-Kit (LSKs). In the chimeras where vehicle-exposed CD45.1 cells competed with vehicle-exposed CD45.2 cells, there was not a significant difference in the frequency of cells identified as LSK (Figure 4A). However, in primary chimeras where vehicle-exposed CD45.1 fetal liver progenitors competed with TCDD-exposed CD45.2 fetal liver chimeras, there was a nearly 50% reduction in the frequency of LSK cells recovered from the bone marrow 8 weeks after the initial transfer (Figure 4B). Furthermore, following the secondary bone marrow transfer, LSK cells from TCDD-exposed CD45.2 cells were severely depleted compared with the vehicle-exposed CD45.1 competitor cells (Figure 4D). Specifically, in the secondary bone marrow chimeras, LSK cells from TCDD-exposed fetuses were reduced by nearly 80% compared with the control competitors (Figure 4E, *p* ≤ 0.01). Unlike the hematopoietic stem and progenitor cell population in the bone marrow, mature effector cells in the peripheral organs could complete their normal maturation program; however, cells from the TCDD-exposed fetuses remained at a competitive disadvantage. For example, in the thymus, although both CD45.1- and CD45.2-derived progenitor cells produced thymocytes in expected proportions, > 50% fewer CD4^+^CD8^+^ thymocytes were derived from the TCDD-exposed CD45.2 cells than from the vehicle-exposed CD45.1 cells (see Figure S3).

### Effects of *in Utero* TCDD Exposure on Cellular Differentiation–Induced Gene Expression Changes

Given the adverse effects of developmental TCDD exposure on the long-term self-renewal of fetal hematopoietic progenitor cells, we analyzed the gene expression changes that occurred between two distinct hematopoietic progenitor cell populations in order to determine how normal differentiation-induced gene expression changes were affected by developmental TCDD exposure. Specifically, we sorted GD 11.5 lineage-negative–c-Kit^+^ fetal hematopoietic progenitor cells based on DCF fluorescence as illustrated by the gating strategy shown in Figure 2; the sorting strategy is further delineated in Figure S4. We chose to compare gene expression between these two cell populations because of the hypothesized difference in self-renewal potential between the DCF^hi^ and DCF^int^ populations (Ito et al. 2006; Jang and Sharkis 2007). Given previous reports that elevated ROS signals a transition to short-term self-renewal and multilineage differentiation, comparing gene expression changes in c-Kit^+^DCF^int^ with those in c-Kit^+^DCF^hi^ was considered to be an innovative approach to determining the effects of *in utero* TCDD exposure on this putative developmental transition. Within these cell populations, we focused our analysis on transcripts known to be involved in Notch and Notch-dependent signal transduction, cellular metabolism, and oxidative stress.

As shown in Figure 5A, the importance of Notch-dependent signal transduction in the transition from long-term (c-Kit^+^DCF^int^) to short-term self-renewal (c-Kit^+^DCF^hi^) was illustrated by decreases in the relative expression of Notch1 and the Notch-dependent transcription factor Hes1 in control fetal cells and an increase in the relative gene expression of the structurally distinct Notch family member protein, Notch4. In comparison, *in utero* TCDD exposure attenuated this differentiation-dependent reduction in Notch signaling (*p* < 0.05) and, furthermore, significantly altered the expression of Hes1 between these two cell populations (*p* < 0.05). The differentiation-induced increase of Notch4 signaling in the c-Kit^+^DCF^int^ to c-Kit^+^DCF^hi^ control transition was abrogated with a significant reduction occurring in TCDD-exposed cells (*p* < 0.05). In addition, we showed that among a panel of metabolic and oxidative stress regulatory genes, TCDD significantly increased pyruvate kinase expression (*p* < 0.05) in c-Kit^+^DCF^hi^ cells relative to c-Kit^+^DCF^int^ cells (Figure 5B), in contrast to the relatively reduced expression observed in controls. Finally, expression of the known TCDD target gene and eNOS regulator GTP-cyclohydrolase (Andreasen et al. 2006; Carney et al. 2006) was decreased by TCDD during this cellular developmental transition (*p* < 0.05).

## Discussion

Given the dual function that hematopoietic stem cells possess to undergo self-renewal and multilineage differentiation for production of the effector cells responsible for immune system function, identification of the impact of developmental environmental exposure on these cells is urgently needed. Moreover, the sensitivity of the immune system to developmental insults that persist throughout life is consistent with the conclusion that a progenitor population present in the fetus is a target of intrauterine environmental exposures. Thus, we believe that we are the first to report experimental evidence that is consistent with a direct effect of prenatal TCDD exposure on impairment of hematopoietic stem cell long-term self-renewal.

Our conclusion that transplacental TCDD has a direct effect on hematopoiesis is based on our studies with AHR-deficient hematopoietic stem cells placed into T-lymphocyte differentiation cultures. Specifically, this experiment examined the contribution of direct AHR activation in the fetus on T-lymphocyte differentiation potential. We report that fetal hematopoietic stem cells from AHR-deficient fetuses were resistant to the inhibitory effects of TCDD. These data suggest that direct activation of the AHR in the fetus is the primary mechanism by which TCDD mediates its developmental immunotoxicity.

Given these data supporting the hypothesis that the developmental immunotoxic effects of TCDD was through direct AHR activation in the fetus, the next objective was to identify the target cell population affected. We found that AHR activation in the fetus increased oxidative stress in hematopoietic progenitor cells. Although activation of the AHR by TCDD is known to affect ROS (Kennedy et al. 2013; Kopf and Walker 2010; Wan et al. 2014), it is unclear from the present study whether this phenomenon occurs because of increased induction of P450 enzymes or because of potential changes in the cell’s metabolic activity. However, induction of the Phase I enzymes Cyp1a1 and Cyp1b1 is not nearly as robust in hematopoietic-derived cells as in other tissues (Ahrenhoerster et al. 2014; Lawrence et al. 1996), which suggests that this change could be attributable to energetic changes occurring in the cell. Hematopoietic stem cells in adult bone marrow maintain low cytoplasmic ROS by producing energy via glycolytic anaerobic respiration (Jang and Sharkis 2007; Simsek et al. 2010). In comparison, the fetal hematopoietic stem cell energetic balance is less well understood and changes throughout development (Imanirad et al. 2014; Orkin and Zon 2008). Nevertheless, it is well established that an elevated oxidative state is detrimental for long-term self-renewal (Iiyama et al. 2006; Ito et al. 2006; Takubo et al. 2013).

To further evaluate the implications of the elevated oxidative state, we sorted fetal hematopoietic progenitors according to DCF, and therefore ROS, levels and conducted a focused analysis of gene expression changes dependent on the developmental stage of the cells and on TCDD exposure. Genes were selected based on their role in Notch signaling, cellular metabolism, and oxidative stress. Notch and Hes1 were selected based on the previously described function during self-renewal whereby decreased Notch activity leads to differentiation (Duncan et al. 2005) and because Hes1 has been identified as a target gene of TCDD (Thomsen et al. 2004). Thus, the decrease in Notch1 and Hes1 expression (Figure 5A) in control c-Kit^+^DCF^hi^ cells compared with their expression in control c-Kit^+^DCF^int^ cells is consistent with a loss of self-renewal potential during the normal process of maturation. In contrast, Hes1 expression was increased in TCDD-exposed c-Kit^+^DCF^hi^ cells compared with that in TCDD-exposed c-Kit^+^DCF^int^ cells, whereas the decrease in Notch1 was significantly smaller in the TCDD-exposed cells than the corresponding decrease in control cells. Notably, Hes1 is a transcriptional repressor involved in a Notch feedback loop that is under strict regulation in stem cells (Guiu et al. 2013), and loss of that strict regulation via TCDD exposure could affect hematopoietic stem cell renewal capacity. Furthermore, the expression of Notch4, which is reported to be involved in hematopoietic stem cell maintenance (Vercauteren and Sutherland 2004), was greater in control c-Kit^+^DCF^hi^ cells than in control c-Kit^+^DCF^int^ cells and was significantly reduced following *in utero* TCDD exposure. Taken together, the altered expression patterns of Notch1, Hes1 and Notch4 in the TCDD-exposed c-Kit^+^DCF^hi^ cells relative to those in their c-Kit^+^DCF^int^ precursors combined with the pronounced shift toward elevated ROS in these fetal cells could be indicative of premature differentiation of the hematopoietic stem cell pool. A potential consequence of this premature differentiation is that it effectively depletes the pool of long-term self-renewing cells at a critical time during development, and the organism cannot recover.

In addition to the effects of TCDD on the Notch pathway, we also measured potential changes in cellular metabolism and oxidative stress regulatory genes caused by elevated ROS. Notably, TCDD induced significantly greater pyruvate kinase (PKM2) expression in cells undergoing maturation from c-Kit^+^DCF^int^ to c-Kit^+^DCF^hi^ than in control cells undergoing the same developmental maturation, suggestive of a transition to a more metabolically active cell with potentially elevated ROS. However, although other metabolic and oxidative stress genes were largely unchanged, these studies do not rule out the potential effects of developmental AHR activation on the activity level of the enzymes encoded by the genes analyzed. Thus, a more comprehensive metabolomic and proteomic approach designed to test the normal energy regulation of these hematopoietic cells and to determine the effects of TCDD may offer novel mechanistic insight into AHR regulation of cellular metabolism in hematopoietic stem cells during critical developmental transitions.

Taken together, by analyzing the gene expression changes that are associated with normal developmental transitions occurring during hematopoiesis, we have identified two potential mechanisms accounting for the loss of self-renewal and the increase in ROS in fetal hematopoietic stem cells. The data showing TCDD-induced alterations of Notch and Hes1 expression combined with the shift in the proportion of cells in c-Kit^+^DCF^int^ to c-Kit^+^DCF^hi^ are suggestive of premature maturation of the hematopoietic stem cell pool, which effectively depletes the number of long-term hematopoietic stem cells. Alternatively, the increased PKM2 expression could be indicative of conversion of the cells from glycolysis to oxidative phosphorylation; the premature occurrence of this conversion during development could have an adverse effect on the establishment of long-term self-renewal in the fetus.

In comparison to the role of the AHR in fetal development, which is just beginning to be understood, it is well established that the AHR acts as an important regulator of hematopoiesis in adult model systems. Specifically, new classes of nontoxic AHR agonists and antagonists indicate a role for the AHR in hematopoietic stem cell expansion and maturation while maintaining multipotential differentiation capacity (Boitano et al. 2010; Carlin et al. 2013; Smith et al. 2013). Other reports have suggested that the AHR maintains hematopoietic stem cells in a quiescent state by acting as a negative regulator of cell proliferation (Gasiewicz et al. 2014; Singh et al. 2009) while maintaining a low intracellular oxidative state (Singh et al. 2014). In support of this model, a possible interpretation of our data is that the cytoplasmic-localized AHR complex has a normal function maintaining low levels of ROS in hematopoietic stem cells. Thus, in the presence of a potent agonist such as TCDD, dissociation of the AHR from its cytoplasmic complex and concomitant nuclear translocation of the AHR removes a ROS regulator from the quiescent hematopoietic stem cell cytoplasm. In possible support of this model, both our own data and data reported by others demonstrate that baseline levels of ROS are elevated in the absence of the AHR. Taken together, our ROS data in both AHR^–/–^ and TCDD-exposed cells along with our gene expression findings suggest a potential mechanism whereby TCDD activation of the AHR in fetal hematopoietic stem and progenitor cells increases Notch-dependent signal transduction in the cell and accelerated maturation. Elevated Notch signaling combined with potentially elevated metabolic activity and the associated increase in ROS adversely affect long-term self-renewal.

Beyond the mechanistic implications for developmental AHR activation in hematopoiesis, the environmental health impact of AHR activation by TCDD on hematopoiesis in the fetus is more sensitive than comparable studies in adult mice. Specifically, the dose of 3 μg/kg TCDD administered to the pregnant dam in the current study was ≥ 10 times lower than the dose used in experiments testing the self-renewal potential of adult bone marrow hematopoietic stem cells (Casado et al. 2011; Sakai et al. 2003). Moreover, the dose used for the present study was well within the 1- to 10-μg/kg TCDD exposures utilized in recent developmental basis of health and disease rodent studies testing cardiomyocyte, epidermal, and immunological end points (Muenyi et al. 2014; Wang et al. 2013; Winans et al. 2015). Collectively, those studies and our own all resulted in fetal exposures in the ppt range, based on measurements showing that only ~ 0.5% of the TCDD administered to a pregnant C57BL/6 dam was transferred to the fetus (Weber and Birnbaum 1985). Although comparisons between rodent studies and human exposures are inherently complicated by differences in pharmacokinetics, gestation, reproductive biology, life-span, and complex mixtures exposures, these *in utero* exposures are within an order of magnitude of the toxic equivalent (TEQ) estimates for several populations across the globe based on food consumption (Hochstenbach et al. 2012; Schecter et al. 2001) or on gas chromatography/mass spectrometry exposure assessment of maternal blood, cord blood, and placenta (Suzuki et al. 2005).

Identification of the hematopoietic system as a sensitive target affected by *in utero* exposures has broad implications for later-life health and disease, given the essential role of hematopoiesis in the formation of the blood and the immune system. Any perturbation of the developing hematopoietic system has the potential to adversely affect a spectrum of later-life blood diseases including anemia, cancer, and immune suppression. It should be noted, however, that developmental TCDD exposure may be complex, with adverse effects occurring in both the hematopoietic and stromal compartments (Boule et al. 2014). Additionally, it cannot be determined from the results of the present study whether exposure to other AHR agonists during development would produce a similar outcome. Nevertheless, these findings should initiate further research to determine the effects of different classes of AHR-active compounds on long-term self-renewal and to identify other environmental factors present in the intrauterine environment that have impacts on the development of the hematopoietic system.

## Conclusions

To our knowledge, we have shown for the first time that developmental exposure to a low dose of TCDD impairs the long-term self-renewal of hematopoietic stem cells. The potential mechanism of this self-renewal impairment is consistent with AHR activation occurring directly in the fetus and is associated with increased ROS levels in fetal hematopoietic stem cells.

## Supplemental Material

(2.6 MB) PDF
