# Supplementary material for: Effects of Developmental Activation of the Aryl Hydrocarbon Receptor by 2,3,7,8-Tetrachlorodibenzo-p-dioxin on Long-term Self-renewal of Murine Hematopoietic Stem Cells
Source: Environ Health Perspect. 2015 Oct 23;124(7):957–65. doi: 10.1289/ehp.1509820 (PMC4937855; doi:10.1289/ehp.1509820)
Supplement: (2.6 MB) PDF [file ehp.1509820.s001.acco.pdf]

Environ Health Perspect
DOI: 10.1289/ehp.1509820

Note to readers with disabilities: *EHP* strives to ensure that all journal content is accessible to all readers. However, some figures and Supplemental Material published in *EHP* articles may not conform to 508 standards due to the complexity of the information being presented. If you need assistance accessing journal content, please contact ehp508@niehs.nih.gov. Our staff will work with you to assess and meet your accessibility needs within 3 working days.

Supplemental Material

Effects of Developmental Activation of the Aryl Hydrocarbon Receptor by 2,3,7,8-Tetrachlorodibenzo-*p*-dioxin on Long-Term Self-Renewal of Murine Hematopoietic Stem Cells

Michael D. Laiosa, Everett R. Tate, Lori S. Ahrenhoerster, Yuhong Chen, and Demin Wang

Table of Contents

Table S1. Gene name, accession number and primer sequence used for qRT^2PCR
Figure S1. Effect of developmental exposure to TCDD on ROS in fetal hematopoietic cells. (A) Expression of hematopoietic lineage markers with the lin- gate used for all analysis for both vehicle and TCDD exposed fetuses shown. (B) Lin- cells were further analyzed for expression of cKit (GD 11.5, not shown), or cKit vs Sca-1 to identify hematopoietic stem and progenitor cells. The number in each gate represents the percentage of cells from each fetus. (C) DCF fluorescence was then analyzed in each cKit vs Sca population for fetal cells from both vehicle-exposed (red shaded histograms) or TCDD-exposed pregnant dams (blue solid line). The putative self-renewing hematopoietic stem cell population is found in the lin-cKit+Sca-1+ population and was thus further analyzed as illustrated in figure 2.
Figure S2. Effect of elevated ROS on apoptosis in fetal hematopoietic progenitor cells on GD 14.5: Cells were stained to identify LSKs, fixed, permeabilized and DNA breakage analyzed by the TdT-dependent nick-end labeling assay using the APO-BrdU kit from Phoenix Flow Systems according to the manufacturers instructions (A) Lin- cells from vehicle or TCDD-exposed pregnant dams were analyzed for cKit and Sca-1. (B) Manufacturer-provided negative control

(blue histogram) was compared with TdT positive control (red histogram) to set the gates for determination of apoptosis. (C) LSK cells from vehicle (blue histogram) is overlaid with LSK cells from TCDD-exposed dams (red histogram). Data were analyzed in FloJo using the population comparison function to conclude that there was not a significant difference between LSK cells obtained from vehicle or TCDD.

Figure S3. Effects of prenatal TCDD exposure on thymocyte differentiation in secondary bone marrow transfers: (A) Flow cytometric representation of thymocytes from the Vehicle to vehicle (left FACS plot) mixed secondary chimeras compared to the Vehicle to TCDD (right FACS plot) secondary chimeras. FACS plots are representative of four mice per group. (B) Percentage of thymocytes derived from each donor population shown in panel A. Data are the mean $\pm$ SEM with 4 mice per group and experiment was repeated once. The white bars represent Vehicle-to-Vehicle chimeras, and Vehicle-to-TCDD chimeras represented by blue bars. Solid bars are the CD45.1+ cells and the striped bars are the CD45.2+ cells. The composition of CD45.1 vs. CD45.2 are comparable to the bone marrow (Figure 3D). (C) Differentiation of thymocytes within the CD4 versus CD8 populations is shown within the CD45.1+ or CD45.2+ gated cells with the vehicle-to-vehicle secondary chimeras shown above the Vehicle to TCDD mixtures. (D) Percentage of thymocytes within each developmental stage is shown with white bars representing chimeras derived from the vehicle-to-vehicle mixture and blue bars representing chimeras derived from vehicle to TCDD mixture. Solid bars represent the CD45.1+ population and striped bars represent the CD45.2+ population. Data are presented as the mean $\pm$ SEM with n = 4 mice per group with a single repeat. (E) Proportion of thymocytes within each developmental stage derived from each donor is shown with white bars representing chimeras derived from the vehicle-to-vehicle mixture and blue bars representing chimeras derived from vehicle to TCDD mixture. Solid bars represent the CD45.1+ population and striped bars represent the CD45.2+ population. Statistical significance determined by Tukey's t test after ANOVA is denoted with an *** for $p \leq 0.01$.

Figure S4. Cell sorting strategy for qPCR analysis. (A) Cells are first gated based on Forward X Side scatter area. (B) Cells are next gated based on viability using Sytox Blue exclusion. (C) Within viable cells, doublet discrimination is accomplished by Forward scatter height X width and (D) Side scatter height x width gating. (E) After doublets are excluded, lineage negative cells are gated. (F) Lin- cells are further restricted by analysis of CD45-cKit+. (G) Two-way cell

sorting gates are then established in order to purify cKit+DCF^{int} and cKit+DCF^{hi} in vehicle (left FACS plot) or TCDD-exposed fetuses (right FACS plot).

Table S1. Gene name, accession number and primer sequence used for qRT^{2}PCR

Name	Accession	5'->3' Forward	5'->3' Reverse
Hairy enhancer of split (Hes-1)	NM_008235.2	tgccagctgatataatggagaa	ccatgataggctttgatgacttt
Delta-like 1 (DL1)	NM_001190703	agcaaacgtgacaccaagtg	taagtgttggggcgatcttc
Jagged 1 (Jag1)	NM_013822	gtgccctggtgtccattc	taggaccgctggcagatg
Notch4	NM_010929	cctgcctgaagagggagag	cagaaatccaggggcaca
Notch1	NM_008714	cagaacaccaatggcagcta	acagttgcgacctgtatagcc
GTP cyclohydrolase (GTPCH1)	NM_008102	gccgcttactcgtccattct	gaacaaggtgatgctcacaca
Superoxide dismutase (SOD2)	NM_013671	tgctctaatcaggacccattg	gtagtaagcgtgctcccacac
carnitine palmitoyltransferase 2 (CPT2)	NM_009949	cagcacagcatcgtaccca	tcccaatgccgttctcaaaat
Pyruvate kinase (PKM2)	NM_001253883	gaagccacacagtgaagcag	tgtgttccaggaaggtgtca
Fatty acid synthase (FASN)	NM_007988.3	ccctgaccaaggtgctgt	gttgtggaagtgcaggttagg
carnitine palmitoyltransferase 1 (CPT1)	NM_013495	tcttcactgagttccgatggg	acgccagagatgccttttcc
tuberous sclerosis 1 (TSC1)	NM_001289575	atggcccagttagccaacatt	cagaattgagggactccttgaag
CD36	NM_001159555	cgggccacgtagaaaacact	cagccaggactgcaccaata
HPRT	NM_013556.2	gtcaacgggggacataaaag	caacaatcaagacattctttcca

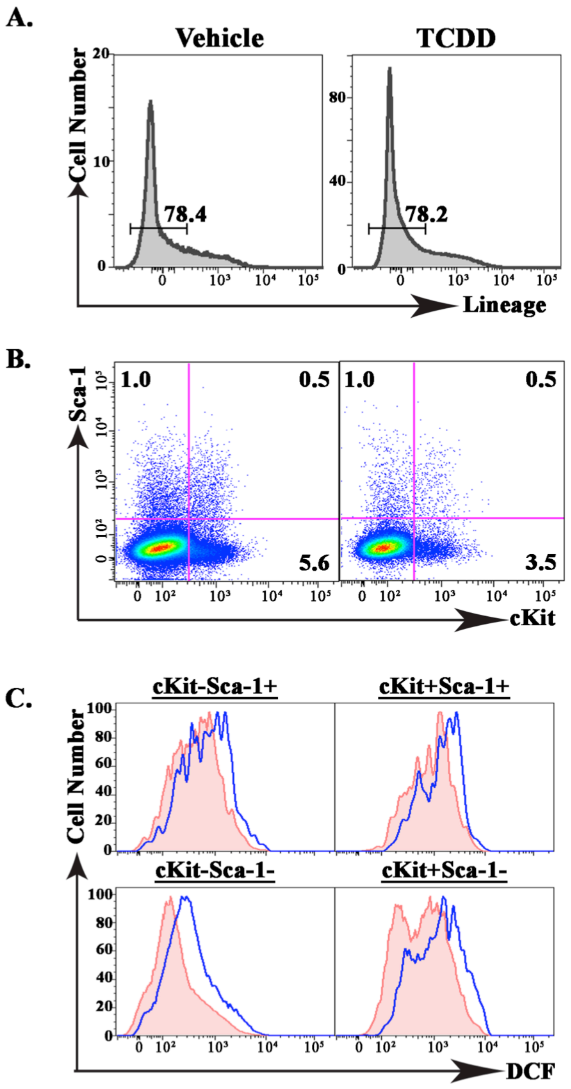

Figure S1. Effect of developmental exposure to TCDD on ROS in fetal hematopoietic cells. (A) Expression of hematopoietic lineage markers with the lin- gate used for all analysis for both vehicle and TCDD exposed fetuses shown. (B) Lin- cells were further analyzed for expression of cKit (GD 11.5, not shown), or cKit vs Sca-1 to identify hematopoietic stem and progenitor cells. The number in each gate represents the percentage of cells from each fetus. (C) DCF fluorescence was then analyzed in each cKit vs Sca population for fetal cells from both vehicle-exposed (red shaded histograms) or TCDD-exposed pregnant dams (blue solid line). The putative self-renewing hematopoietic stem cell population is found in the lin-cKit+Sca-1+ population and was thus further analyzed as illustrated in figure 2.

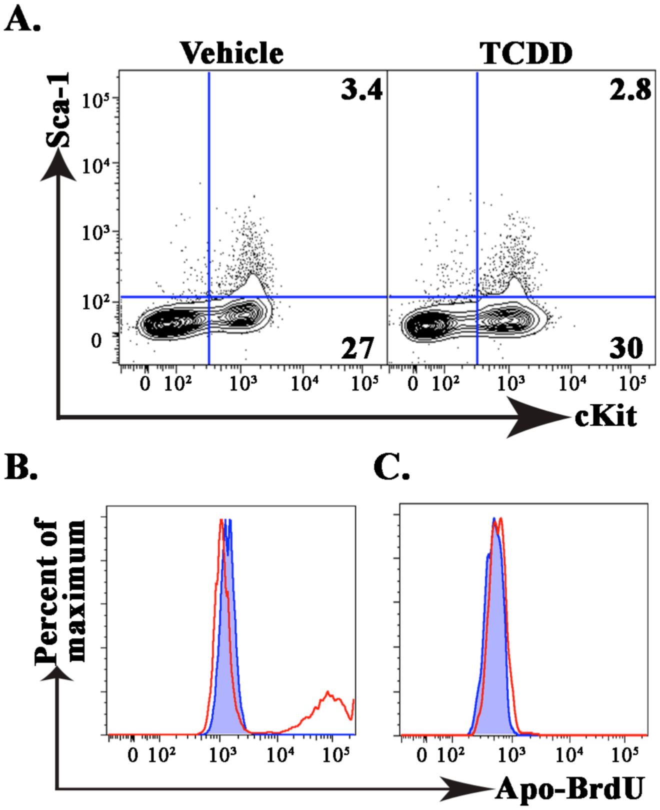

Figure S2. Effect of elevated ROS on apoptosis in fetal hematopoietic progenitor cells on GD 14.5: Cells were stained to identify LSKs, fixed, permeabilized and DNA breakage analyzed by the TdT-dependent nick-end labeling assay using the APO-BrdU kit from Phoenix Flow Systems according to the manufacturers instructions (A) Lin- cells from vehicle or TCDD-exposed pregnant dams were analyzed for cKit and Sca-1. (B) Manufacturer-provided negative control (blue histogram) was compared with TdT positive control (red histogram) to set the gates for determination of apoptosis. (C) LSK cells from vehicle (blue histogram) is overlaid with LSK cells from TCDD-exposed dams (red histogram). Data were analyzed in FloJo using the population comparison function to conclude that there was not a significant difference between LSK cells obtained from vehicle or TCDD.

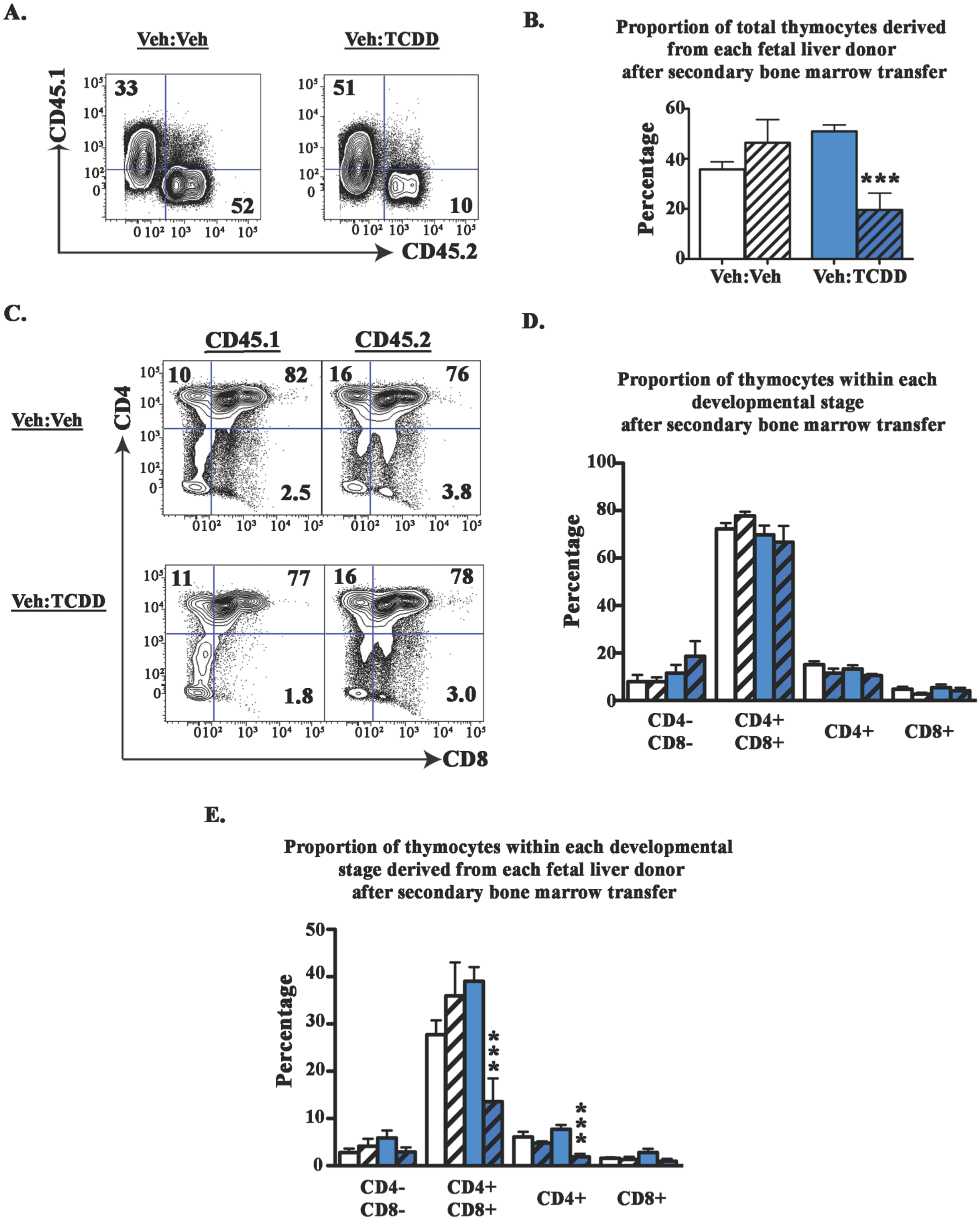
A.
Veh:Veh
Veh:TCDD
CD45.1
CD45.2
33
52
51
10
B.
Proportion of total thymocytes derived from each fetal liver donor after secondary bone marrow transfer
Percentage
Veh:Veh
Veh:TCDD
***
C.
CD45.1
CD45.2
Veh:Veh
Veh:TCDD
CD4
CD8
10
82
2.5
16
76
3.8
11
77
1.8
16
78
3.0
D.
Proportion of thymocytes within each developmental stage after secondary bone marrow transfer
Percentage
CD4- CD8-
CD4+ CD8+
CD4+
CD8+
E.
Proportion of thymocytes within each developmental stage derived from each fetal liver donor after secondary bone marrow transfer
Percentage
***
***
CD4- CD8-
CD4+ CD8+
CD4+
CD8+

Figure S3. Effects of prenatal TCDD exposure on thymocyte differentiation in secondary bone marrow transfers: (A) Flow cytometric representation of thymocytes from the Vehicle to vehicle (left FACS plot) mixed secondary chimeras compared to the Vehicle to TCDD (right FACS plot) secondary chimeras. FACS plots are representative of four mice per group. (B) Percentage of thymocytes derived from each donor population shown in panel A. Data are the mean $\pm$ SEM with 4 mice per group and experiment was repeated once. The white bars represent Vehicle-to-Vehicle chimeras, and Vehicle-to-TCDD chimeras represented by blue bars. Solid bars are the CD45.1+ cells and the striped bars are the CD45.2+ cells. The composition of CD45.1 vs. CD45.2 are comparable to the bone marrow (Figure 3D). (C) Differentiation of thymocytes within the CD4 versus CD8 populations is shown within the CD45.1+ or CD45.2+ gated cells with the vehicle-to-vehicle secondary chimeras shown above the Vehicle to TCDD mixtures. (D) Percentage of thymocytes within each developmental stage is shown with white bars representing chimeras derived from the vehicle-to-vehicle mixture and blue bars representing chimeras derived from vehicle to TCDD mixture. Solid bars represent the CD45.1+ population and striped bars represent the CD45.2+ population. Data are presented as the mean $\pm$ SEM with n = 4 mice per group with a single repeat. (E) Proportion of thymocytes within each developmental stage derived from each donor is shown with white bars representing chimeras derived from the vehicle-to-vehicle mixture and blue bars representing chimeras derived from vehicle to TCDD mixture. Solid bars represent the CD45.1+ population and striped bars represent the CD45.2+ population. Statistical significance determined by Tukey's t test after ANOVA is denoted with an *** for $p \leq 0.01$.

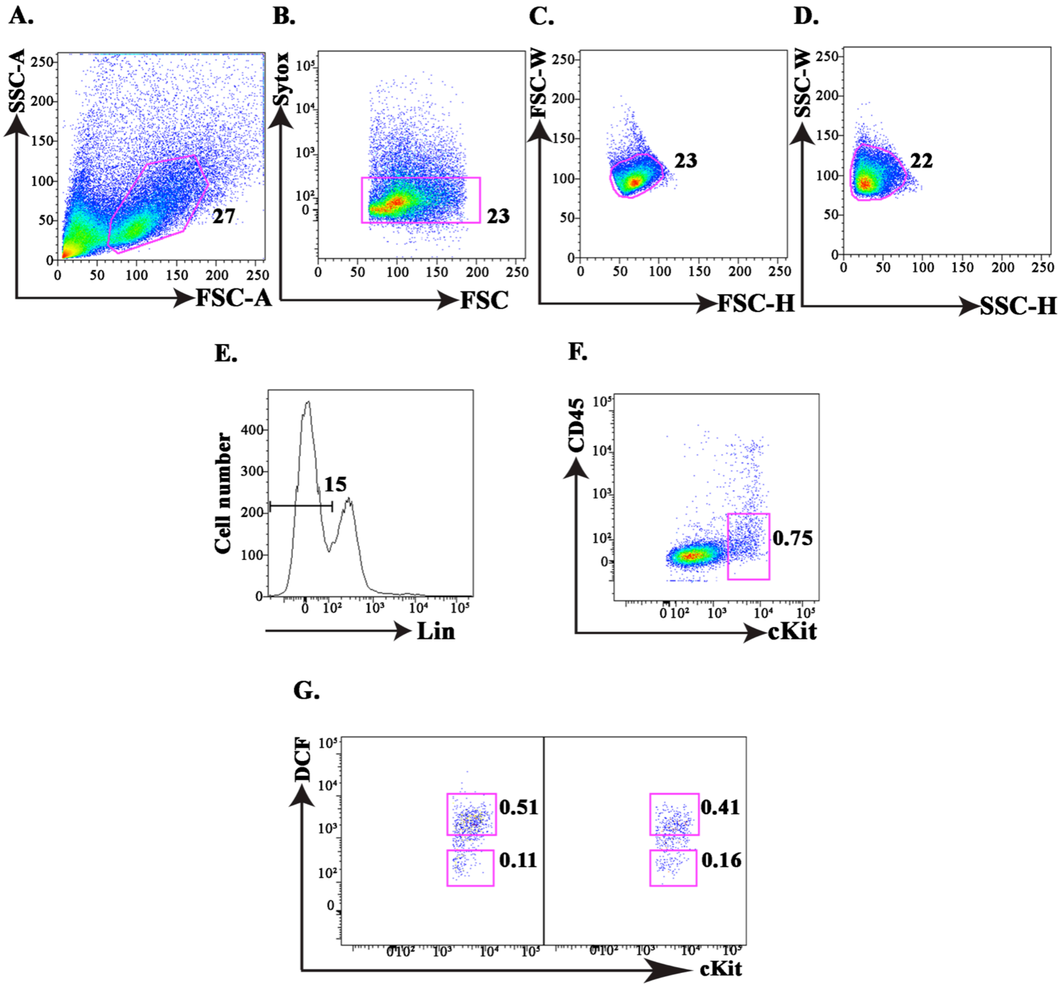

Figure S4. Cell sorting strategy for qPCR analysis. (A) Cells are first gated based on Forward X Side scatter area. (B) Cells are next gated based on viability using Sytox Blue exclusion. (C) Within viable cells, doublet discrimination is accomplished by Forward scatter height X width and (D) Side scatter height x width gating. (E) After doublets are excluded, lineage negative cells are gated. (F) Lin- cells are further restricted by analysis of CD45-cKit+. (G) Two-way cell sorting gates are then established in order to purify cKit+DCF^{int} and cKit+DCF^{hi} in vehicle (left FACS plot) or TCDD-exposed fetuses (right FACS plot).
